# Xenocortical mice: what can we learn, and how should this research progress?

**DOI:** 10.1093/jlb/lsag026

**Published:** 2026-10-27

**Authors:** Nita Farahany, Anil K Seth

**Affiliations:** Robinson O. Everett Professor of Law and Philosophy, Duke University, Durham, NC, US; School of Engineering and Informatics and Centre for Consciousness Science, University of Sussex, Brighton BN1 9QJ, UK; Canadian Institute for Advanced Research, Program on Brain, Mind, and Consciousness, Toronto, Canada

## Abstract

A new study by researchers at Stanford University describes the development of ‘xenocortical mice’: mice with a cortex comprised almost entirely of human-derived neurons. Their groundbreaking research has the potential to enable medical breakthroughs for a range of neurological and psychiatric disorders. The researchers offer detailed analysis of the neurophysiological and behavioral properties of these chimeric mice, comparing them with both wild-type control mice, and ‘apallial’ mice lacking most of their cortex. Here, we outline some key findings from the study and ask what these findings tell us about the capacity for consciousness in these mice, as well as their potential for human-like properties of cognition or conscious experience. Based on this discussion, we summarize the ethical and regulatory landscape relevant to chimeric animals and lay out an ethically-informed research roadmap to guide future research in this important area.

## I. OVERVIEW

Researchers at a Stanford laboratory have developed a mouse model whose cerebral cortex is primarily made up of human neurons, to enable better research on the human brain.^1^ The cortex, the outermost layer of gray matter in the brain that handles sensory processing, motor control, and decision-making, was genetically suppressed in these mice before birth, resulting in what the researchers called an ‘immuno-deficient apallial mouse’. The researchers then filled the area that would have been occupied by the mouse cortex with ‘human stem-cell derived cortical organoids’, human brain tissue that had been grown from pluripotent stem cells. As these ‘xenocortical’ mice developed, the cortical grafts became integrated with the host nervous system.

These xenocortical mice behaved much as other mice do, exploring mazes and remembering which parts of a maze they have just seen. But they also showed behavioral differences from both fully intact mice and those with their cortex depleted (the apallial mice), differences the researchers measured and reported without offering an opinion on what they may mean for how we should ethically regard the resulting animals.

Here we raise two questions to help guide the next steps in the field. First, how should we think about these chimeric mice, and is there any reason to believe they have any human-like cognitive competences or capacities for experience? And second, who should decide what may be done as this research progresses, how should these decisions be made, and based on what evidence? (Similar questions—and some suggestions—have been made at an earlier stage of this research^2^).

In their paper, the Stanford team calls for engagement among ethicists, researchers, patient representatives, and regulatory bodies to ensure responsible advancements following this study. We agree, and we believe that engagement should happen now, before the next set of experiments build upon this one.

## II. THE STANFORD STUDY

Living human brain tissue is extremely difficult to study *in vivo*, in part due to understandable ethical restrictions on human experimentation. Yet there are many potential benefits to doing so, perhaps most notably in developing new treatments for neurological disorders. A promising approach has been to utilize human stem-cell-derived cortical organoids (hCOs), either in isolation, in preparations consisting of multiple organoids (assembloids), or transplanted into host animals.^3^ The Stanford group previously demonstrated the power of this approach by developing a potential therapeutic for Timothy Syndrome—a rare but devastating genetic disorder—reversing the pathophysiology in all three contexts: organoids, assembloids, and transplantations.^4^

Transplantation experiments are particularly significant because (unlike organoids and assembloids) they enable advanced circuit and in particular behavioral readouts from the host animals, which in turn allows more informative interpretations and comparisons. However, transplanting hCOs into host animals is also the most technically challenging because the human-derived tissue must developmentally compete with host tissue, and rodent tissue develops more quickly. This procedure also raises novel ethical issues, as we discuss below.

This is why the new study from Kaganovsky et al. is potentially game-changing. They reasoned that transplantation might be more successful if host animals could be genetically manipulated to lack (most of) their own cortex, thereby avoiding the challenges posed by competition between the native cortex and the implanted hCOs.

They were right. By implanting hCOs into genetically modified ‘apallial’ mice, which lack large swathes of their native cortex and hippocampus (~98 per cent), they created ‘xenocortical’ (XCX) mice. At the time of transplantation, the hCOs were 30–60 days old, and the mice were just a few days postnatal—prior to the critical period in mouse development when anatomical and functional connectivity becomes established.^5^ Three months following transplantation, in seven of the animals measured, the implanted hCOs made up 91.9 per cent of the combined cortical tissue volume–brain regions the researchers called ‘XCX grafts’ (Fig. 2E in Kaganovsky et al*.*; subsequent figure references also refer to this article). These XCX grafts integrated tightly within the host nervous system, with connections extending to the thalamus, pallidum, and other subcortical and paleocortical areas. Strikingly, histological analyses identified a cluster of neurons with similarities to human Von Economo neurons (VENs) (Extended Data Fig. 5h,i). This is noteworthy because these large, spindle-shaped layer V neurons are not found in rodents: they are instead associated with a small number of large-brain species besides humans (great apes, elephants, cetaceans, Old World monkeys).^6^ VENs have also been linked to social and emotional cognition, as well as affective aspects of conscious experience.^7^

In further analyses, Kaganovsky et al*.* used calcium imaging and electrophysiology to identify waves of activity that propagated across the entire XCX graft in about 100 ms, as well as local field potential bursts. These findings suggest the presence of electrically active and functionally integrated neural networks, reminiscent of patterns of activity reported in mid-gestational human cortical tissue.^8^ (A useful analogy here might be to think of the XCX graft as like an orchestra tuning up, rather than performing a symphony.)

As mentioned, compared to organoids and assembloids, xenocortical mice uniquely allow analysis of behavior. Here, Kaganovsky et al*.* report many fascinating results. Perhaps most strikingly, both xenocortical mice and control mice—but not apallial mice—performed above chance at a ‘Y maze’ task requiring memory of previously explored environments. At the same time, xenocortical mice differed from control mice in how they freely explored open environments, and in how well they learned associations between sensory stimuli (Fig. 4). These analyses are just the starting point, but they already reveal that xenocortical mice have sophisticated behavioral repertoires that depart from those of typical mice. Finally, the authors found that depriving xenocortical mice of oxygen damaged the implanted cortex and led to deficits in gait that were not seen in control or apallial mice under the same hypoxic conditions. This highlights the clinical potential of their approach, for example in developing novel models of cerebral palsy.

## III. CONSCIOUSNESS AND HUMAN-LIKE PROPERTIES

There is no doubt that Kaganovsky et al*.* have pushed forward the frontiers of biology and lighted the path to medical breakthroughs with their xenocortical mice. Yet, as they also recognize, there is a great deal ethically at stake.

One topic notable for its absence in their paper is any discussion of consciousness. This omission is understandable given the empirical uncertainties and the ethical complexities involved. Yet their transplantation process immediately raises the question of whether their mice might have minds, and perhaps conscious experiences, that are inflected by the human-derived parts of their cortex. To paraphrase Thomas Nagel: *what is it like to be a xenocortical mouse*?

As things stand now, the answer may well be: nothing at all. The cortex-wide waves of neural activity observed in XCX grafts look very different from the complex desynchronized activity patterns characteristic of cortical activity in waking conscious states in humans and in wild-type mice at 3 months postnatal (let’s assume that such mice are indeed conscious). As just mentioned, they have more in common with activity patterns reported in midgestational human cortical tissue.

But this answer is neither certain nor final. For one thing, while most current theories of consciousness emphasize a dependence (in mammals) on cortical activity,^9^ some theories suggest that brainstem and other subcortical structures might be sufficient for some basic forms of conscious experience.^10^ On these theories, conscious states in xenocortical mice remain plausible.

There is also considerable uncertainty about when consciousness emerges in human development, with one article putting a plausible lower bound for the emergence of consciousness at the end of the second trimester of development (~6 months), based on estimated cortical readiness for thalamocortical integration.^11^ Notably, Kaganovsky et al*.* terminated their investigations at 177 days (~6 months) following cellular differentiation of the hCOs, which, based on transcriptional evidence, corresponds roughly to this time period (Extended Data Fig. 4). However, it is difficult to compare these time points between humans and xenocortical mice, given the mismatched developmental trajectories involved. As mentioned above, substantial thalamocortical connectivity is already in place in the xenocortical mice 3–4 months postnatally, though at this time point these mice still lack the ventral forebrain cells that are important for large-scale thalamocortical network function.

Importantly, there is nothing in principle to prevent xenocortical mice from developing further (though, as Kaganovsky et al*.* note, the different developmental rates of native brain tissue and hCO cortex might put limits on eventual integration). More mature xenocortical mice might display additional neurophysiological features, such as populations of inhibitory interneurons (which may require cotransplanting a forebrain organoid to form an assembloid *in vivo*) and the emergence of mature laminar structures and distinctive cortical areas, that might collectively make consciousness (of some kind) more plausible.

More developmental time might also enable more sophisticated behavioral assays of consciousness. The xenocortical mice showed deficits in trace conditioning, which is relevant because trace conditioning has been associated with consciousness in other animals,^12^ though apallial mice showed the same deficit, so this assay may track the missing native cortex rather than the XCX graft (Extended Data Fig. 9h-j). But even if so, there is still the general challenge that, beyond the gold standard of verbal report in human beings, no single test is reliably diagnostic of the presence of consciousness across different species.^13^ This challenge becomes even harder when extended to the novel creations of synthetic biology.

One response is to adapt and apply batteries of tests to xenocortical mice, with candidate tests selected by criteria of being applicable and interpretable (testing for consciousness via verbal responses fails both criteria). This battery could include oddball responses for stimuli that are locally regular but globally deviant,^14^ and unlimited associative learning.^15^ The more closely the xenocortical mice align with performance by wild-type mice, the more evidence there is for the presence of at least some kind of (at least mouse-like) consciousness. A reasonable strategy here would be to apply these tests at stages of development when substantial changes in neuroanatomical organization are observed, and/or when the transplantation method itself is adapted (eg, implanting assembloids rather than single organoids).

One existing finding in Kaganovsky et al*.* deserves closer attention: the xenocortical mice spent less time at the center of the dark-activity chamber than either the control or the apallial mice. They also spent less time rearing (standing on two legs) than the control mice, although not less than the apallial mice. Both these behaviors are conventionally interpreted as suggestive of anxiety-like states. If these states have conscious components, welfare considerations obviously become significant.

This is still the easy part. It seems plausible to assume that given enough developmental time, and assuming consciousness in control wild-type mice, xenocortical mice will have conscious experiences too. But will these experiences have human-like properties? And will xenocortical mice have human-like cognitive abilities? For these questions, we can only speculate. The observed behavioral differences between xenocortical and control mice in open-field behavior do not yet indicate any specific human-like properties.

It’s first worth noting that human neurons can be much more complex than rodent neurons. For example, human pyramidal neurons have more independent compartments than the rodent equivalents.^16^ They also have unusually complex dendritic integration capabilities not yet observed in rodents.^17^ These features might underlie an expanded repertoire of capacities and transformations that these neurons can accomplish.

Even more suggestive is the presence of neurons reminiscent of VENs in the XCX grafts. The presence of these neurons suggests the possibility that sufficiently developed xenocortical mice might have conscious experiences that share properties with other VEN-rich species, including humans. The role of VENs in social and affective processes is particularly notable here. Could xenocortical mice experience emotional states that have human-like properties? If these mice are to be used as model organisms for psychiatric disorders, the possibility needs to be considered.

Another (though possibly impractical) approach to the above question is to look for distinctively human signatures of perceptual processing. Dehaene and colleagues have argued that human beings are unusually sensitive to geometric regularities like right angles and symmetry (though this ability may be shared by other primates).^18^ If the relevant perceptual paradigms could be adapted to xenocortical mice (and assuming they have a suitably organized visual cortex), this would be a particularly powerful method for exploring whether these mice have non-rodent aspects of perceptual cognition and perhaps consciousness. If they do, this in turn raises the prospect that xenocortical transplantations represent a new kind of ‘gain of function’ manipulation in which animals gain cognitive and perhaps consciousness-related abilities unavailable to their wild-type cousins.

Altogether, nothing observed thus far suggests the emergence of new, non-rodent functions in the xenocortical mice. But if future studies reveal good reasons to think that xenocortical mice (or subsequent chimeric models) do have distinctively human experiences or traits, then there would also be good reasons to worry about the ethical mandate for creating and/or experimenting on them.

## IV. ETHICAL GUIDANCE AND LEGAL MISMATCH

This brings us to our second question: who should decide how far this novel transplantation research is permitted to progress, how should these decisions be made, and based on what evidence? While these are not new questions, they take on a new urgency in light of this research.^19^ In 2018, one of us asked, together with 16 other colleagues, whether brain tissue ‘that might appear to have conscious experiences or subjective phenomenal states’, would ‘deserve any of the protections routinely given to human or animal research subjects’.^20^ The following year, writing with Tim Bayne and Marcello Massimini, the other one of us asked in a different paper whether even disembodied organoids may under some conditions give rise to ‘islands of awareness’, and if so how we might detect them.^21^ One relevant consideration was whether embodied behavior—absent in organoids—might be a necessary condition for consciousness. That condition has now been met.

Back in 2018, the kind of cortical transplantation described by Kaganovsky et al. was still remote. Nevertheless, we still addressed ethical issues raised by the transplantation of human cells into host animals, including rodents, a feat that had already been accomplished in more limited ways by injecting human stem cells into early-stage rodent and pig embryos.^22^ We recognized that this category might introduce novel welfare interests in the host animal and create human-animal blurring such that ‘which kinds of chimera are permitted … should ultimately be made on a case-by-case basis–taking into account the risks, benefits, and people’s diverse sensitivities’. We urged that consent for donor cells more specifically address their use in the development of brain organoids and the later possibility of transplantation. Each concern applies with even greater force here, with chimeras whose cortex is almost entirely made of human-derived cells.

The eight years since have produced substantial ethical and policy guidance, and we would like to emphasize that the Stanford research carefully followed existing ethical norms and legal constraints. Guidelines from the International Society for Stem Cell Research (ISSCR) issued in 2021 distinguish transplants, in which human cells are introduced into the brain of the postnatal animal, from chimeras, in which stem cells are injected prenatally or through embryonic integration and then allowed to develop in parallel with the host. The Stanford study produced transplants, where the guidance is comparatively permissible. The ISSCR guidelines describe postnatal transplants as entities that ‘will generally be of little concern’, while treating transplants into the central nervous system with greater care, calling for baseline behavioral data, pilot studies, and ongoing welfare monitoring of the host animal by animal research committees. A targeted update in 2025 left these provisions unchanged.^23^ The guidelines do not specify what capacities should be measured or what findings should prompt a pause in the research and say little about how to assess changes in the host animal’s capacity for experience, or how the prospect of such changes should inform decisions to create the animal to begin with.

By contrast, the National Academies of Sciences, Engineering and Medicine 2021 report (on organoids, transplants, and chimeras) focused on the capacities of the models themselves.^24^ It found that decisions about how this research should be overseen ‘depend in large part on the possibility that the animal host will have altered capacities’ and that the capacities that are often raised—pain and consciousness—‘are difficult to define or measure’.^25^ Because ‘it appears likely that rodents do have emotions’, it explained, assessing the ‘emotional capacity in transplants or chimeras would have to be based not on acquisition of emotional responses, but rather on assessment of alterations in the emotional capability of the transplant or chimera compared with an unmanipulated host’.^26^ The report said it would be increasingly important to ‘devise methods for assessing’ the ‘characteristics and capacities’ of these models, for assessing pain, and for developing ‘acceptable methods for assessing’ the presence of consciousness, noting that the most informative tests may be the ones that show some difference between altered and unaltered animals of the same species.

Like the ISSCR guidelines, some US state laws also draw a line between integration of human cells embryonically versus postnatally, prohibiting certain human-animal hybrids. Louisiana RS14:89.6 makes it a crime to ‘knowingly create or attempt to create a human-animal hybrid’, which includes ‘[a] nonhuman life form engineered such that it contains a human brain or a brain derived wholly or predominately from human neural tissues’.^27^ Arizona has a nearly identical law, Ariz. Rev. Stat. § 36–2312.^28^ Both statutes provide an exception for xenotransplantation of human tissues into recipient animals when they are not ‘animal embryos’ and occur postnatally, as the Stanford study did here, with postnatal transplant of hCOs into the mice after they were born. The line drawn at the embryonic stage appears to rest on the assumption that early introduction of human cells may lead to more extensive and unpredictable contributions to the host animal, including to its brain and germline, while later transplantation may be more confined. The Kaganovsky et al. study gives some reason to question this assumption. Their method for xenocortication, of genetically depleting the host cortex before transplantation, shows that a postnatal transplant can result in an animal whose cortex is nearly entirely human-derived, such that the timing of transfer may be of less importance than previously thought to how we should ethically regard the resulting animal.^29^

The National Academies report also describes a three-tiered ethical research review process, but its examples would put postnatal neural transplants in mice in a category that doesn’t require additional review, and it doesn’t define the criteria that would move research into a higher tier of review.

The report of a Stanford and Dana Foundation-led meeting at Asilomar in November 2025^30^ cautioned against premature prohibitions and recommended developing an ongoing international process that could respond rapidly to scientific change and proposed review triggers that could prompt additional review.^31^ We agree that a process is urgently needed to guide the research forward, and note that no existing guidance specifies the triggers necessary for heightened review, or how evidence that transplantation has altered the animal’s experience, or given it any human-like capacities, should factor into ethical review. The tension here is that the relevant evidence can only come from creating the animals to begin with. This tension points toward a strategy of staged escalation in host animals with review checkpoints defined in advance.

## V. A ROADMAP FORWARD

Considering how their research should progress, the Stanford researchers called for close collaboration between ‘ethicists, researchers, patient representatives and regulatory bodies’, to ensure the ‘responsible advancement of transplantation models and support their application to investigating human biology and developing therapeutics’. They also list developments that should trigger ethical guidance without saying what should happen when one is reached, including ‘studies at later stages of maturation, cortical layering or gyrification, physiological representation of excitatory and inhibitory neurons, and mature network properties’. We propose that each should trigger the review process described below, within an ethical roadmap that includes at least the following elements:

### V.A. Independent Review

The study was approved by Stanford’s stem cell oversight and animal care committees, who focus on the provenance of the cells and the care and welfare of the animals. Neither is designed to evaluate capacities that may change in the animal or detect or measure consciousness, and existing guidelines leave it to the discretion of the institution on whether to bring in additional expertise to these reviews. The Stanford researchers chose to do so voluntarily, engaging an independent (uncompensated, arm’s-length) external advisory board, on which one of us served.

We propose making this step mandatory before a chimeric or transplanted model is carried past specific thresholds. Some thresholds are features of a study design known in advance, such as maturation of the neural tissue *in vivo* beyond the stage carried out in the present Kaganovsky et al*.* study, the use of assembloids rather than organoids, embryonic hosts, or selection of a new host species. Others are observed outcomes, such as any indication that a transplanted animal has acquired capacities beyond those typical of the host species, or that transplantation has negatively impacted its welfare relative to wild-type animals of the same species. An independent review should involve animal care and use committees and ideally would engage a standing panel of the sort that the Asilomar recap report (mentioned earlier) identifies, with diverse and broad expertise.

### V.B. Standard ‘System Card’ Reporting

Independent review requires evidence from at least three points in the process. The first is a protocol record that is filed before any human neural cells are transferred into a host animal. Second, the monitoring reports at pre-specified intervals during the study. The third is a standardized report that should accompany any published results.

Before transplantation into a host animal, researchers should create a standardized protocol record that records the source and age of the organoid or assembloid, the host species, strain, and its age at transplantation, the planned endpoint of the study (along with its justification), and the predicted maturation stage of the graft at that endpoint.

During any approved study, there should be pre-specified fixed intervals at which welfare and behavioral measures are taken and reported to oversight review committees, which should have the authority to require changes to the study, to pause it, or to call for its termination.

At publication, studies should adopt a standardized reporting system that provides the maturation stage of the graft at transplantation and the endpoint, its cellular composition, whether the transplanted material is a single organoid or a more complex assembloid, any network activity, and the results of a standardized battery of behavioral metrics. This battery should include assays of affective state and welfare, and assays relevant to the capacity for consciousness, and to the presence of human-like properties of cognition.

A useful analogy here is provided by the ‘system cards’ that accompany each new release of a frontier artificial intelligence model,^32^ which document the system as deployed, together with the safeguards and the behaviors tested in the system. An *in vitro* organoid is more easily characterized, but the same cells transplanted into an intact mouse, a cortically depleted one, or a different host species may result in different animals with different behavioral outcomes. Unlike a system card, these organoid system cards must begin before transplantation rather than upon release of a new animal model. Reporting of this kind would facilitate the comparison of data across studies, and it would also enable the field to make evidence-based decisions about where the ethical thresholds should apply.

### V.C. Specific Consent

The cell donors in the Stanford study specifically consented to *in vivo* transplantation of their cells, which should become the norm in studies like this one. Consent by donors should be specific for the *in vivo* use of their cells (or for very specific applications) rather than blanket consent, because a donor who agrees to contribute cells for research may not have contemplated that those cells would be induced into a pluripotent state and become a living organoid, or transplanted into a host animal. Consent for governance could alternatively allow donors to consent to a standing committee that owes them a duty to consider their best interests in approving novel uses of donated cells.^33^ The specific type of consent should be included on the standard reporting ‘model card’ for the study.

### V.D. Slow Escalation to Other Species

There is much to learn scientifically and ethically from transplantation in rodents, and many questions remain unanswered about how to ethically regard the chimeric mice we already have. Moving from rodents to pigs or to non-human primates may be scientifically and clinically useful to better approximate the human brain, but a better approximation brings more complex ethical concerns about the capacities, or potential gained capacities by those chimeric animals. A relevant consideration here is that other species, in particular non-human primates, may have host nervous systems and developmental trajectories that would facilitate deeper integration of hCO transplants. The burden should be on the researcher to show what a host closer to humans in its developmental timetable would make possible that a rodent model cannot. A separate question is whether there is a social mandate for this research, which is part of the broader deliberative process that the Asilomar report calls for and we agree is warranted.

## VI. CONCLUSION

Nothing the researchers measured in their pioneering study suggests the xenocortical mice have any human-like capacities, nor that they have gained functions that wild-type mice do not already have. However, this is not the end of a research program, but a continuation of a research trajectory that shows increasingly more sophisticated integration of human neural cells into non-human hosts. This research offers both greater promise for addressing unmet needs in human health and suffering, as well as new opportunities for studying the brain basis of cognition and consciousness, including aspects that might be distinctively human. But it also opens up many new gray areas and unanswered ethical questions that should be addressed before proceeding onwards to chimeric and transplanted models that more closely approximate the human brain.

## Footnotes

1 Konstantin Kaganovsky et al., *Developmental Xenocortication Using Human-Derived Organoids in Mice*, 658 Nature 430 (2026).

2 Julian Savulescu & Tsutomu Sawai, *Animus: Human-Embodied Animals*, 50 J. Med. Ethics 725 (2024); Henry T. Greely et al., *Thinking About the Human Neuron Mouse*, 7 Am. J. Bioethics 27 (2007).

3 Sergiu P. Pașca et al., *A Framework for Neural Organoids, Assembloids and Transplantation Studies*, 639 Nature 315 (2025).

4 Xiaoyu Chen et al., *Antisense Oligonucleotide Therapeutic Approach for Timothy Syndrome*, 628 Nature 818 (2024).

5 Zoltán Molnár & Kenneth Y. Kwan, *Development and Evolution of Thalamocortical Connectivity*, 16 Cold Spring Harbor Perspect.  Biol. a041503 (2024); Joshua A. Gordon & Michael P. Stryker, *Experience-Dependent Plasticity of Binocular Responses in the Primary Visual Cortex of the Mouse*, 16 J. Neurosci. 3274 (1996).

6 Ivan Banovac et al., *Von Economo Neurons – Primate-Specific or Commonplace in the Mammalian Brain?*, 15 Front. Neural  Circuits 714611 (2021).

7 Franco Cauda et al., *Evolutionary Appearance of von Economo’s Neurons in the Mammalian Cerebral Cortex*, 8 Front  Hum  Neurosci 104 (2014); A.D. (Bud) Craig, *How Do You Feel—Now? The Anterior Insula and Human Awareness*, 10 Nat. Rev. Neurosci. 59 (2009).

8 Anna R. Moore et al., *Spontaneous Electrical Activity in the Human Fetal Cortex In Vitro*, 31 J. Neurosci. 2391 (2011).

9 Anil K. Seth & Tim Bayne, *Theories of Consciousness*, 23 Nat. Rev. Neurosci. 439 (2022).

10 Bjorn Merker, *Consciousness Without a Cerebral Cortex: A Challenge for Neuroscience and Medicine*, 30 Behav. Brain  Sci. 63 (2007); Mark Solms, The Hidden Spring: A Journey to the Source of Consciousness (2021).

11 Hugo Lagercrantz, *The Emergence of Consciousness: Science and Ethics*, 19 Semin. Fetal  Neonatal  Med. 300 (2014); *see also*, Joel Frohlich et al., *Not with a "Zap" but with a "Beep": Measuring the Origins of Perinatal Experience*, 273 NeuroImage 120057 (2023).

12 Paula Droege et al., *Trace Conditioning as a Test for Animal Consciousness: A New Approach,* 24 Anim. Cogn. 1299 (2021); Robert E. Clark & Larry R. Squire, *Classical Conditioning and Brain Systems: The Role of Awareness*, 280 Science 77 (1998).

13 Tim Bayne et al., *Tests for Consciousness in Humans and Beyond*, 28 Trends  Cogn. Sci. 454 (2024).

14 Lynn Uhrig et al., *Cerebral Responses to Local and Global Auditory Novelty Under General Anesthesia*, 141 NeuroImage 326 (2016).

15 Zohar Z. Bronfman et al., *The Transition to Minimal Consciousness Through the Evolution of Associative Learning*, 7 Front. Psychol. 1954 (2016). For a review of candidate tests, *see* Bayne et al., *supra* note 13.

16 Sapir Shapira et al., *Biophysical and Computational Insights from Modeling Human Cortical Pyramidal Neurons*, 19 Front. Neurosci. 1579715 (2025); Ido Aizenbud et al., *Dendritic Morphology and Synaptic Nonlinearities Enhance Functional Complexity in Human Cortical Neurons*, 123 Proc. Nat’l  Acad. Sci. e2533168123 (2026).

17 Albert Gidon et al., *Dendritic Action Potentials and Computation in Human Layer 2/3 Cortical Neurons*, 367 Science 83 (2020).

18 Mathias Sablé-Meyer et al., *Sensitivity to Geometric Shape Regularity in Humans and Baboons: A Putative Signature of Human Singularity*, 118 Proc. Nat’l  Acad. Sci. e2023123118 (2021); Jialin Li et al., *Continuity in Geometric Intuition Between Humans and Monkeys*, 123 Proc. Nat’l  Acad. Sci. e2532934123 (2026).

19 Savulescu & Sawai, *supra* note 2; Greely et al., *supra* note 2.

20 Nita A. Farahany et al., *The Ethics of Experimenting with Human Brain Tissue*, 556 Nature 429 (2018).

21 Tim Bayne et al., *Are There Islands of Awareness?*, 43 Trends  Neurosci. 6 (2020).

22 Jun Wu et al., *Interspecies Chimerism with Mammalian Pluripotent Stem Cells*, 168 Cell 473 (2017).

23 Int’l Soc’y for Stem Cell Rsch., *Guidelines for Stem Cell Research and Clinical Translation* (ver. 1.2, 2025).

24 Nat’l Acads. of Scis., Eng’g & Med., *The Emerging Field of Human Neural Organoids, Transplants, and Chimeras: Science, Ethics, and Governance* (2021), https://doi.org/10.17226/26078 (Note that both the current authors contributed to this report).

25
*Id.* at 8.

26
*Id.* at 41.

27 La. Stat. Ann. § 14:89.6 (2009).

28 Ariz. Rev. Stat. Ann. § 36-2312 (2010).

29 Savulescu & Sawai, *supra* note 2.

30 Bruce Goldman, *Considering Assembloids and Organoids*, Stanford Medicine Magazine (2026), https://stanmed.stanford.edu/conference-ethics-assembloids-organoids/ (accessed Oct. 13, 2026).

31 Brie Linkenhoker, *Toward Global Oversight of Human Neural Organoid and Assembloid Research: Report from the 2025 Asilomar Conference on Ethical and Societal Implications of Neural Organoids, Assembloids, and Their Transplantation* (2026), https://neuroscience.stanford.edu/sites/default/files/2026-09/Full%20report%20-%202025%20Asilomar%20Conference%20on%20Oversight%20of%20Neural%20Organoids_0.pdf (accessed Oct. 13, 2026).

32 NHI Mgmt Grp. Editorial Team, *What Is the Difference Between a System Card and a Model Card?*, NHI Mgmt Grp., https://nhimg.org/faq/what-is-the-difference-between-a-system-card-and-a-model-card/ (last updated Sept. 10, 2026).

33 Sarah N. Boers & Annelien L. Bredenoord, *Consent for Governance in the Ethical Use of Organoids*, 20 Nat. Cell  Biol. 642 (2018).

